# The Lymphoproliferative Disorders: Handbook of diagnosis, investigation and management

**DOI:** 10.1038/sj.bjc.6690402

**Published:** 1999-05-01


*British Journal of Cancer* (1999) **80**(3/4), 625–626
© 1999 Cancer Research Campaign
Article no. bjoc.1998.0402

### Book reviews

### The Lymphoproliferative Disorders: Handbook of diagnosis, investigation and management

Child, Jack and Morgan: Publication details, pp. 383, £49.95, ISBN 0412580306. Edward Arnold (Publishers) Ltd: London, 1998.

In the last 5 years there have been major advances in the understanding of malignant lymphoma and other lymphoproliferative diseases, particularly at the molecular level. Along with this, diagnostic precision has increased dramatically and many distinct clinico-pathological entities can now be readily identified. The revised European American Lymphoma (REAL) classification, shortly to be superseded by the WHO classification of lymphoma, incorporates many of these new findings and forms the basis of this book which, according to the authors, is intended for those undertaking higher specialist training in haematology, pathology or oncology, those in established posts who require a review of the subject, and research workers, specialist nurses and laboratory staff involved in the fields of haematopathology. The book divides broadly into two parts, the first of which covers principles of diagnosis and treatment and the second of which deals with specific entities within the lymphoproliferative disorders.

Part 1 comprises easy to read and accessible accounts of the structure of the lymphoid system, laboratory methods for diagnosis and classification and then moves on to cover staging and imaging and principles of therapy of lymphoproliferative disorders, finishing with a chapter on classification. The chapters on lymphoid structure and laboratory methods are particularly strong providing excellent summaries of the subjects. The laboratory methods included in Chapter 2 include conventional morphology, cytochemistry and immunohistochemistry but move on to give clear and well-illustrated accounts of other methodologies including flow cytometry, Southern blotting, polymerase chain reaction, in situ hybridization, etc. These chapters are particularly useful and will prove especially valuable to clinicians who wish to have a basic grasp of modern diagnostic techniques in lymphoma pathology.

The chapter on staging is nicely divided by anatomical site and gives a reasonably up to date and concise account of available radiological techniques, although is possibly a little misleading in places. For example, the role of routine chest radiography is somewhat understated, particularly for Hodgkin's disease, in which, at present, assessment of mediastinal masses on chest X-ray remains a major factor in therapeutic decisions. The role of magnetic resonance imaging as a means of assessing disease viability in residual masses and staging the bone marrow is overstated since there have been conflicting reports of the value of this technique and, unfortunately, the chapter presents relatively little data on the use of Gallium scanning and none on the use of PET scanning, which is an emerging technique in lymphomas.

The chapter on principles of therapy begins with an excellent description of apoptopic pathways and the way in which currently available treatments can modify these. It is a welcome change from the standard account of the action of cytotoxic drugs and radiotherapy etc.

Section 2 of the book covers the specific lymphoproliferative disorders, divided up roughly along the lines of the REAL classification, although the authors acknowledge that it was not possible to adhere strictly to this. Nevertheless, this provides a novel approach to the accounts of these various lymphoma entities.

Each of these chapters take a roughly similar approach to the groups of diseases being discussed. Each provides a thorough description of the diagnosis, pathology and molecular pathology of the disorders followed by, in most cases, a concise but relatively brief account of treatment. This section of the book is particularly strong in its coverage of pathology, diagnosis and underlying molecular mechanisms, and is heavily biased in this direction. Accounts of management of the diseases are relatively brief and will be inadequate for those who wish to gain an insight into some of the complexities of clinical management. For example, the chapter on Hodgkin's disease provides an elegantly written account of the various sub-types of Hodgkin's disease, including lymphocyte predominant nodular Hodgkin's disease and then the various types of classical Hodgkin's with an extensive description of their pathology, the nature of the Reed–Sternberg cell, etc. However, treatment issues in Hodgkin's disease are condensed into only approximately three pages and fail to convey the complexity of the treatment decisions in this disease, the balance between disease control and long-term toxicity and the many ongoing clinical trials. Similarly, in this chapter and the chapter on the peripheral B-cell lymphoproliferative disorders, the role of high-dose therapy in stem cell transplantation is somewhat understated.

The general presentation of the book is very attractive. It is broken into small sections interspersed with useful 'take home messages' in bold type which stand out clearly and emphasize many important points. There are a very large number of line drawings, which are particularly useful. All of the photomicrographs, however, are half-tones. Presumably this is in order to keep the cost of the book down, but it is obviously very difficult to convey modern immunohistochemical techniques in half-tone illustrations.

A relatively large number of computerized tomography scans are reproduced in the book and, unfortunately, many of these are of rather low quality. A small number of colour illustrations are included, although it is not entirely clear why the authors chose these particular entities as colour plates.

Each section of the book ends with a list of key references. There are significant omissions from these. For example, the chapter on peripheral B-cell diseases does not contain a reference to the International Prognostic Index, to the South West Oncology Group randomized trial in aggressive non-Hodgkin's lymphoma, or to the Parma protocol, all of which are landmark papers in this entity. Similarly, the Hodgkin's disease chapter does not acknowledge the contribution of the EORTC Lymphoma Trials Group studies, or include a reference to the CALGB randomized trial which established ABVD as standard therapy in this disease.

Despite these criticisms, many clinicians and others involved in the treatment of haematological malignancy will find this book extremely useful. It will be of particular use to those wishing to gain a clearer insight into the diagnosis and pathogenesis of lymphoproliferative disorders, but will probably be inadequate for those wishing to have a full account of the clinical issues surrounding these conditions.

*JW Sweetenham*
*Medical Oncology Unit,*
*University of Southampton,*
*Southampton SO16 6YD*

### Clinical Cancer Genetics

K Offit: Publication details, pp. 419, ISBN 0-4711-4655-2, Wiley Liss: UK, 1998.

Within a week of receiving this book for review, it had become one of my first-line reference texts and I recommended that we buy more copies for our Department. Kenneth Offit has used his wide experience in the field of cancer genetics to produce a broad-based text covering all aspects of cancer genetics. Not only is the scientific basis of cancer genetics explained in detail, but the practical problems of using it in the clinic setting explored in chapters looking at genetic testing and the management of patients with cancer, reproductive counselling for cancer patients and their families and finally the psychological, ethical and legal issues in cancer risk counselling. The part of genetics I have always found the most challenging has been the complex risk calculations that one has to embark on when you are looking at syndromes with variable age of onset and non-penetrance, such as HNPCC and BRCA1 and BRCA2. I found the chapter on quantitative methods in cancer risk assessment one of the clearest written I have ever read.

There are two long chapters covering the everyday management of specific cancer predisposition syndromes. One covers the common hereditary cancers (breast, ovarian, colon and prostate), and the other the commoner cancer predisposition syndromes. I found the information given was up to date and accurate. Reading the text as a geneticist there were useful discussions about patient details that we are not so familiar with such as the significance of the pathology of different types of tumours.

The book ends with a series of appendices. The only criticism I have of the book is Appendix A, which gives a Table of inherited disorders that may predispose to cancer listed by organ system. The idea behind this is useful in that if you look up the malignancy that you are interested in, it then gives you the organ system in which you will find syndromes with these tumours. I have used this several times in the clinic setting and found the searching laborious. I have also found the information given on a particular condition to be different depending on what body system it is listed in. In at least one case (Neurofibromatosis type 1) the information given is conflicting. This is, however, a minor criticism. Appendix B gives a list of laboratories in the USA which offer the various cancer genetic tests and their approximate price. It also provides very useful Internet resources. The final appendix gives Tables of familial risk for breast, ovarian and colon cancer.

The text is indispersed with relevant case histories which helps to reinforce the points made in the general text.

Who should buy this book? I really have no hesitation in advising that all those involved in cancer genetic counselling on a regular basis should purchase a copy. For those who are less often involved, but want a reference text to occasionally dip into, I would be tempted to delay purchase of a new cancer genetic textbook until the second edition of Hodgson and Maher (*A Practical Guide to Human Cancer Genetics*) becomes available. Comparing Offit's book with the first edition of Hodson and Maher's book is not really fair as this came out in 1993 and so is now quite out of date. A new edition is promised in the near future. As Offit wrote his book based on experience in an American cancer centre, the advantage of the Hodgson and Maher text would be that it is written with an up to date perspective on cancer genetic counselling in the UK.

*SM Huson,*
*Department of Clinical Genetics,*
*Oxford Radcliffe Hospital,*
*The Churchill Hospital,*
*Old Road, Headington,*
*Oxford OX3 7LJ*

*© Cancer Research Campaign 1999*

